# Vitamin B12 deficiency may play an etiological role in atrophic glossitis and its grading: A clinical case-control study

**DOI:** 10.1186/s12903-022-02464-z

**Published:** 2022-10-28

**Authors:** Guan-Ying Chen, Zhi-Qun Tang, Zhe-Xuan Bao

**Affiliations:** Department of Oral Medicine, Affiliated Stomatology Hospital of Guangzhou Medical University, Guangdong Engineering Research Center of Oral Restoration and Reconstruction, Guangzhou Key Laboratory of Basic and Applied Research of Oral Regenerative Medicine, 195 Dongfeng West Road, 510182 Guangzhou, China

**Keywords:** Atrophic glossitis, Vitamin B12 deficiency, Grading, Etiological factor

## Abstract

**Background:**

Existing studies have reported the significant association between atrophic glossitis (AG) and hematinic deficiencies, including iron, folate and vitamin B12 deficiency. However, these findings were inconsistent. AG can be graded as partial or complete atrophy. It is still unclear whether hematinic deficiencies are associated with the grading of AG.

**Methods:**

236 AG patients and 208 sex- and age-matched healthy controls were enrolled in this study. Hematological tests including complete blood count, and serum levels of folate, ferritin and vitamin B12 were performed. The AG group was divided into those with partial AG and those with complete AG according to the extent of papillary atrophy. Statistical analysis was performed to assess whether hematinic deficiencies are risk factors for AG and its grading.

**Results:**

Compared with the healthy controls, AG patients had significantly higher frequencies of vitamin B12 deficiency (68.22%), ferritin deficiency (13.98%) and anemia (21.61%). The differences in hematinic deficiencies and anemia between AG patients and healthy controls changed according to gender and age. The frequencies of serum vitamin B12 deficiency and anemia in the complete AG subgroup were significantly higher than those in the partial AG subgroup. Logistic regression analysis revealed that vitamin B12 deficiency and anemia were significantly correlated with AG and its grading. The AG patients with vitamin B12 deficiency responded well to supplement therapy.

**Conclusion:**

AG could be an important clinical indicator for potential vitamin B12 deficiency, especially when the degree of tongue atrophy more than 50% and complete atrophy. Vitamin B12 deficiency might play an etiological role in the development of AG.

## Background

The tongue is considered a mirror of general health, and can provide clinical clues to many systemic diseases [1–3]. Atrophic glossitis (AG) is manifested by partial or complete loss of lingual papillae on the dorsal surface of the tongue [3, 4]. The relationship between nutritional deficiencies and AG was first described in 1975 in the first US National Health and Nutritional Examination Survey [5]. It is now widely accepted that AG may be a marker of nutritional deficiency [2–6].

Existing studies have reported the significant association between AG and hematinic deficiencies, including iron, folate and vitamin B12 deficiency [3, 7]. Possible underlying mechanisms are that vitamin B12 and folate play crucial roles in DNA synthesis and cell metabolism. The epithelial cells of lingual papillae have a rapid turnover, resulting in their sensitivity to deficiencies of these two vitamins [4, 8]. In addition, patients with iron deficiency or iron deficiency anemia, have reduced oxygen carrying capacity to the dorsal surface mucosa of the tongue [8].

However, the findings from these previous studies were inconsistent. Sun et al. found that AG patients had a significantly higher frequency of iron and vitamin B12 deficiency or anemia, but not folate deficiency, compared to healthy controls [7]. By contrast, an earlier study proposed that tongue mucosa atrophy was a significant clinical finding indicating decreased serum folate level, but had no significant association with serum vitamin B12 level [9]. Unexpectedly, a previous population survey revealed that elderly people with AG had significantly higher serum levels of vitamin B12 than those who had normal tongue [4]. Therefore, further studies are needed to investigate or validate the association between AG and hematinic deficiencies.

Based on the extent of lingual papillae involvement, tongue atrophy is graded as partial or complete atrophy [10]. The profile of hematinic deficiencies based on the grade of AG is still unclear. Thus, it would be helpful to further understand the etiology of AG and whether hematinic deficiencies could affect the grading of AG, which has not yet, to our best knowledge, been reported.

The aim of this study was to determine the association between hematinic deficiencies and AG. Moreover, the profile of hematinic deficiencies based on the grade of AG was also examined. Statistical analysis was performed to assess whether hematinic deficiencies are risk factors for AG and its grading.

## Methods

The AG group consisted of 236 patients (61 men and 175 women, age range 18–86 years, median 61 years, QR: 52, 71 years). According to previous studies, AG was diagnosed when lingual papillae atrophy affected more than 50% of the tongue [4, 11]. A group of 208 sex- and age-matched healthy controls (63 men and 145 women, age range 19–88 years, median 58.5 years, QR: 52.25, 75 years) were included for analysis. All AG patients were diagnosed consecutively from March 2017 to May 2021 at the Department of Oral Medicine, the Affiliated Stomatology Hospital of Guangzhou Medical University, China. The exclusion criteria were based on previous studies [3, 7]. Specifically, patients with autoimmune diseases (such as Sjogren’s syndrome, systemic lupus erythematous and rheumatoid arthritis), severe liver or kidney diseases, diabetes mellitus and other systemic diseases were excluded. Besides, patients with other concomitant oral mucosal diseases such as oral lichen planus (OLP), traumatic lingual ulcer, oral submucous fibrosis (OSF) and pemphigus vulgaris (PV) were also not enrolled. Healthy controls, who had either dental caries or chronic periodontal diseases but did not have any oral mucosal or systemic diseases, were recruited from other departments of the same hospital. The present study was approved by the ethics committee of our hospital.

## Laboratory methods

After obtaining written informed consent from the participants, and after overnight fasting, blood samples were collected from each participant. Serum levels of folate, ferritin and vitamin B12 in all participants were determined using routine methods in the Department of Clinical Laboratory of our hospital. In addition, a complete blood count was carried out. Serum folate, vitamin B12 and ferritin deficiencies were defined as a serum level under the lower limit of the normal range. The reference serum folate and vitamin B12 levels are 4.0–18.7 ng/mL and 180–914 ng/L, respectively. The normal accepted range of serum ferritin level is 11.0–306.8 ng/mL for women and 15–336.2 ng/mL for men. Anemia was defined as a hemoglobin (Hb) level less than the cutoff value (male < 12 g/dL and female < 11 g/dL).

## Clinical grading of AG

**Figure 1:**
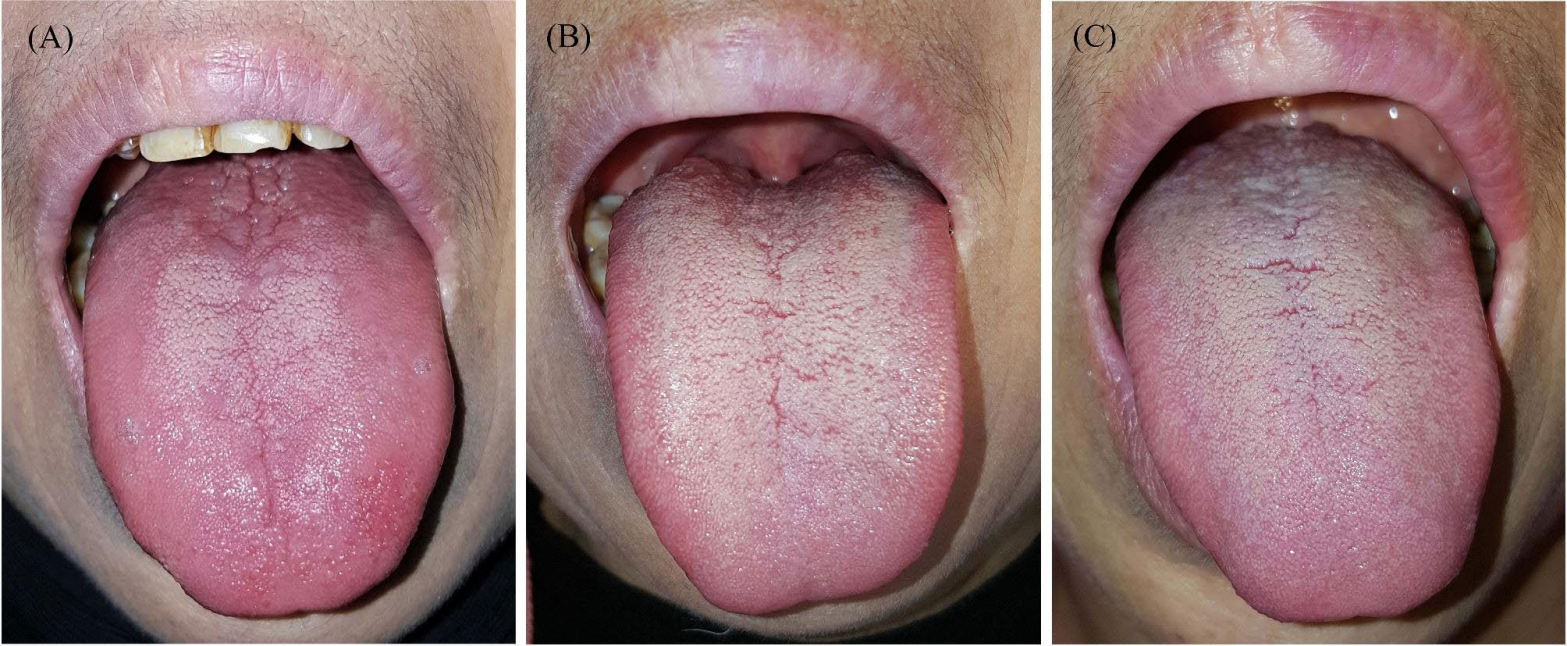
An example of partial AG. A 58-year-old woman with the chief complaint of a burning sensation on the tongue for six months. She was easily fatigued for several months and denied any systemic diseases. Her serum vitamin B12 level was 72 ng/L (normal range 180–914 ng/L) and serum levels of folate, ferritin and hemoglobin were all within the normal range. (A) Physical examination revealed partial atrophy of the tongue; (B) complete resolution after 1 month of supplement therapy; (C) the therapeutic effect was still evident during follow-up at 6 months.

**Figure 2:**
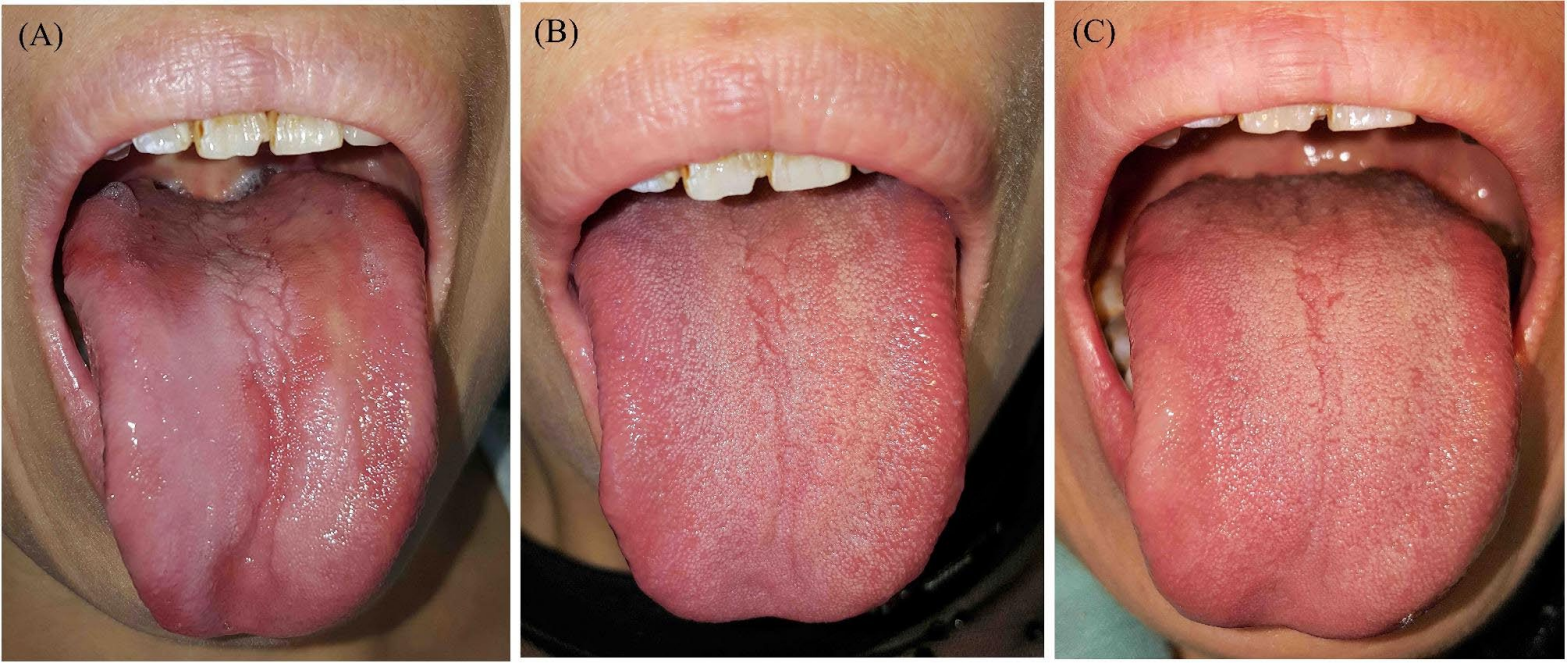
An example of complete AG. A 46-year-old woman with the chief complaint of glossodynia for approximately one year. She denied any systemic diseases. Her serum levels of vitamin B12 and ferritin were 94 ng/L (normal range 180?914 ng/L) and 4.3 ng/mL (normal range 11.0?306.8 ng/mL), respectively. She was also diagnosed with anemia based on a hemoglobin level of 10.8 g/dL (the cutoff value for a female is 11 g/dL). (A) Physical examination revealed complete tongue atrophy, with a glossy appearance; (B) complete resolution after 1 month of supplement therapy; (C) the therapeutic effect was still evident during follow-up at 6 months.

According to the extent of papillary atrophy and loss, the AG group was divided into two subgroups: partial AG (absence of lingual papillae on the dorsum of the tongue was more than 50%, but not complete, Fig. 1 A) and complete AG (dorsal tongue showed complete absence or flattening of papillae, leaving a glossy, smooth tongue appearance, Fig. 2 A). The grading of AG was performed by two authors (GY Chen and ZX Bao) independently. When grading was inconsistent between these two specialists, a consensus was reached by discussion with a third researcher (ZQ Tang).

## Supplement therapy of AG

According to the hematological examination results, corresponding nutritional supplements were given to the patients. Patients with folate deficiency and vitamin B12 deficiency were treated with oral folate tablets (5 mg/d) and mecobalamin (1500 µg/d), respectively, for at least 1 month. Oral ferrous succinate (200–400 mg/d) and vitamin C (300–600 mg/d) were given to patients for 3 months, if ferritin deficiency was present.

### Data analysis

Statistical analysis of the data was performed with SPSS software, version 22.0 (SPSS Inc., Chicago, IL, USA). The Wilcoxon rank sum test was used to compare the differences in age. The male-to-female ratio, the frequencies of serum folate, vitamin B12, and ferritin deficiency and anemia were compared using the chi-square test. According to the results of the chi-square test, multivariate binary logistic regression analysis was used to estimate the association of potential risk factors with AG and its severity, respectively. A *P*-value less than 0.05 was considered statistically significant.

## Results

### Hematinic deficiencies in AG patients compared with healthy controls

The frequency of overall hematinic deficiencies (at least one deficiency in serum folate, ferritin and vitamin B12) was 76.27% (180/236) in AG patients vs. 11.54% (24/208) in healthy controls, and the difference was very significant (*P* < 0.001, Table 1). Similarly, the frequencies of serum vitamin B12 deficiency, serum ferritin deficiency and anemia were all significantly higher in AG patients than in healthy controls (*P* < 0. 001, *P* = 0.035 and *P* < 0. 001, respectively). With regard to serum folate deficiency, a borderline significant difference with a *P*-value of 0.053 was observed between AG patients and healthy controls (Table 1).

**Table 1:** Hematinic deficiencies in the AG patients compared with the healthy controls

	AG patients	Healthy controls	χ^2^	***P***
Overall hematinic deficiencies	76.27% (180/236)	11.54% (24/208)	186.537	< 0.001
Serum folate deficiency	4.67% (11/236)	1.44% (3/208)	3.751	0.053
Serum vitamin B12 deficiency	68.22% (161/236)	3.37% (7/208)	197.709	< 0.001
Serum ferritin deficiency	13.98% (33/236)	7.69% (16/208)	4.456	0.035
Anemia	21.61% (51/236)	3.85% (8/208)	30.080	< 0.001

### Hematinic deficiencies in AG patients based on gender and age

Compared with healthy male controls, male AG patients had higher frequencies of hematinic deficiencies and anemia, but only serum vitamin B12 deficiency and anemia were statistically significant (*P* < 0.001 and *P* = 0.001, respectively, Table 2). Similarly, female AG patients also had significantly higher frequencies of serum vitamin B12 deficiency and anemia compared with healthy female controls (both *P* < 0.001, Table 2).

**Table 2:** The differences of Hematinic deficiencies between AG patients and healthy controls based on gender and age

	Male				Female				<60 years				≥ 60 years			
	AG patients	Healthy controls	χ^2^	***P***	AG patients	Healthy controls	χ^2^	***P***	AG patients	Healthy controls	χ^2^	***P***	AG patients	Healthy controls	χ^2^	***P***
Serum folate	6.56%	3.17%	0.770	0.380	4.0%	0.69%	3.565	0.059	3.51%	2.27%	0.338	0.561	5.74%	0	4.520	0.033
deficiency	(4/61)	(2/63)			(7/175)	(1/145)			(4/114)	(3/132)			(7/122)	(0/76)		
Serum vitamin	80.33%	0	83.670	< 0.001	64.0%	4.83%	118.864	< 0.001	63.16%	3.79%	100.270	< 0.001	72.95%	2.63%	93.231	< 0.001
B12 deficiency	(49/61)	(0/63)			(112/175)	(7/145)			(72/114)	(5/132)			(89/122)	(2/76)		
Serum ferritin	3.28%	0	2.099	0.147	17.71%	11.03%	2.824	0.093	25.44%	10.61%	9.330	0.002	3.28%	2.63%	0.067	0.796
deficiency	(2/61)	(0/63)			(31/175)	(16/145)			(29/114)	(14/132)			(4/122)	(2/76)		
Anemia	16.39%	0	11.234	0.001	23.43%	5.52%	19.410	< 0.001	25.44%	3.03%	26.207	< 0.001	18.03%	5.26%	6.694	0.010
	(10/61)	(0/63)			(41/175)	(8/145)			(29/114)	(4/132)			(22/122)	(4/76)		

Whether older or younger than 60 years of age, AG patients all had significantly higher frequencies of serum vitamin B12 deficiency and anemia compared with healthy controls (both *P* < 0.001 for vitamin B12, *P* < 0.001 and *P* = 0.010 for anemia, respectively, Table 2).

### The difference in age, gender and hematinic deficiencies based on the grading of AG

According to the grading standard, the partial AG subgroup consisted of 140 patients (28 men and 112 women, age range 18–84 years) and the complete AG subgroup consisted of 33 men and 63 women, age range 31–86 years). The statistical analysis revealed significant gender and age differences between the two subgroups (*P* = 0.013 and *P* < 0.001, respectively, Table 3). The frequencies of serum vitamin B12 deficiency and anemia in the complete AG subgroup were significantly higher than those in the partial AG subgroup (*P* = 0.001 and *P* = 0.003, respectively, Table 3).

**Table 3:** The difference of age, gender and hematinic deficiencies based on the grading of AG

	AG patients		***P***
	Partial subgroup	Complete subgroup	
Men-to-women ratio	28:112	33:63	0.013 ^a^
Age (median, QR)	58 (49.25, 67.75)	64.5 (57,74)	< 0.001 ^b^
Serum folate deficiency	4.29% (6/140)	5.21% (5/96)	0.741 ^a^
Serum vitamin B12 deficiency	60.00% (84/140)	80.21% (77/96)	0.001 ^a^
Serum ferritin deficiency	17.1% (24/140)	9.4% (9/96)	0.091 ^a^
Anemia	15% (21/140)	31.3% (30/96)	0.003 ^a^

QR: Quartile range

^a^ Chi-square test

^b^ Wilcoxon’s rank-sum test

### Logistic regression analysis of risk factors for AG and its severity

Based on the results of logistic regression analysis, the potential risk factors for AG included serum vitamin B12 deficiency (*P* < 0.001, OR = 57.247, 95% CI, 25.446–128.789) and anemia (*P* = 0.041, OR = 2.910, 95% CI, 1.047–8.085). However, the correlation between serum folate or ferritin deficiency and AG was not significant (*P* = 0.057 and *P* = 0.151, respectively, Table 4). In AG patients, the logistic regression analysis revealed that age, gender, serum vitamin B12 deficiency and anemia were all significantly correlated with the grading of AG (Table 4).

**Table 4:** Logistic regression analysis of risk factors for AG and its grading

Dependent variable	Variable	P-value	OR	95% CI	
				Lower	Upper
AG	Serum folate deficiency	0.057	4.293	0.960	19.197
	Serum vitamin B12 deficiency	< 0.001	57.247	25.446	128.789
	Serum ferritin deficiency	0.151	1.871	0.796	4.398
	Anemia	0.041	2.910	1.047	8.085
The grading of AG	Gender	0.011	0.433	0.228	0.824
	Age	0.001	2.592	1.452	4.630
	Serum vitamin B12 deficiency	0.029	2.029	1.073	3.836
	Anemia	0.002	3.081	1.534	6.190

### The effects of vitamin B12 supplement therapy

All AG patients with vitamin B12 deficiency responded well to supplement therapy (Figs. 1 and 2). After one month of vitamin B12 supplement therapy, lingual papilla atrophy resolved partially or completely (Figs. 1B and 2B). 112 patients with vitamin B12 deficiency at baseline completed at least six months of follow-up. The therapeutic effect was still evident during follow-up (Fig. 1 C, 2 C), partial recurrence was only observed in 15 cases.

## Discussion

The present study demonstrated that AG could be an important clinical indicator of potential vitamin B12 deficiency, especially when the degree of tongue atrophy is more than 50% or complete atrophy is present. Vitamin B12 deficiency is a health problem both in developed and developing countries [12–14]. However, it is difficult to diagnose vitamin B12 deficiency at an early stage as the early signs and symptoms are usually subtle and nonspecific [9, 15]. AG might be the only initial sign and precedes other systemic manifestations of vitamin B12 deficiency [9]. Due to its convenience, examination of the tongue is of great value in clinical practice [6]. Hence, dentists can contribute to the early diagnosis of vitamin B12 deficiency.

Sun et al. found that the frequency of vitamin B12 deficiency were only 7.4% in a group of 176 AG patients [7] and 5.3% of 1064 AG patients [3]. In this study, the frequency of vitamin B12 deficiency in AG patents was almost 70%. One reason for this discrepancy is that the diagnostic criteria applied were different. The percentage of tongue papillary atrophy was not defined and patients with less than 50% were also enrolled in previous studies [3, 7]. In the present study, patients were diagnosed with AG only when lingual papillae atrophy was more than 50%, which might contribute to more clearly identify the association between hematinic deficiencies and AG [4, 11].

In this study, the frequency of vitamin B12 deficiency in AG patients was significantly higher than that in healthy controls. In the two subgroups of AG, vitamin B12 deficiency was significantly more common in complete AG patients than in partial AG patients. Moreover, logistic regression analysis revealed that vitamin B12 deficiency was significantly associated with AG and its grading, respectively. In the previous study, the number of complete AG patients was only 6 (3.41% of 176 patients) [7]. By contrast, 96 complete AG patients (40.68% of 236 patients) were enrolled in our study. The stricter diagnostic criteria, together with the higher proportion of complete AG patients, could explain the higher frequency of vitamin B12 deficiency in our study. Based on our findings and comparisons with previous studies, it is reasonably assumed that vitamin B12 deficiency might play an etiological role in the development of AG. The optimal response to vitamin B12 supplementation in our study also confirmed this view.

Consistent with previous study [7], we also found that AG patients had a significantly higher frequency of serum ferritin deficiency and anemia than healthy controls. Similarly, a higher frequency of serum folate deficiency, but not with statistical significance, was also observed in AG patients. However, a population-based study suggested that atrophic changes in tongue was significantly associated with decreased serum folate levels [9]. Notably, all the enrolled participants in the population-based study were 70 years or older. Interestingly, we also found that, AG patients had significantly higher frequency of serum folate deficiency than healthy controls when older than 60 years of age. But the statistical significance of this difference disappeared when younger than 60 years. Therefore, it might be plausible to conclude that serum folate deficiency might be much more common in elderly patients with AG. Significant variations in hematinic deficiencies across different genders and age groups had been demonstrated in patients with recurrent aphthous stomatitis (RAS) and OLP [16, 17]. In the present study, we demonstrated that the frequency of serum ferritin deficiency changed significantly according to the age of AG patients, which is consistent with the findings on RAS and OLP [16, 17]. Moreover, we found that the statistical results for anemia were highly matched to those of vitamin B12 deficiency, both in the chi-square test and logistic regression analysis, which is easily understood as anemia in most patients was directly caused by vitamin B12 deficiency.

It should be noted that a description of the color of tongue atrophy, such as pink, red or magenta, proved to be subjective and the consistency was low among clinicians [10]. In addition, the color did not have any significance in AG grading and did not affect the accuracy [10]. Therefore, only the extent of lingual papillae atrophy was considered in the diagnosis of AG and its grading in the present study. Here, two well trained oral medicine specialists assessed tongue atrophy independently in order to minimize subjective errors. If there was any controversy regarding the diagnosis of AG, a third specialist participated in the discussion. For a more objective evaluation of the exact extent of mucosal atrophy, one theoretically feasible method is the application of a non-invasive imaging device and/or medical image analysis software. For example, linked color imaging (LCI) is a recently developed image-enhanced endoscopy system and can clearly distinguish the border of mucosal atrophy under various conditions of gastritis [18]. It might be possible to apply LCI, with some modifications if necessary, to determine the extent of tongue atrophy.

Here, a minority of AG patients had suboptimal therapeutic outcomes, which may have been the result of other coexisting etiological factors, such as candidiasis and xerostomia [19, 20]. Although we excluded the individuals with Sjogren’s syndrome and diabetes mellitus, we did not perform the examination of the tongue for *Candida* and salivary secretion test, which are limitations of our study. Kimori H et al. demonstrated that high *Candida* count and low salivary secretion were closely associated factors for the risk of development of AG [19]. And there is higher prevalence of *Candida* colonization in the individuals with AG than those without AG in xerostomia patients who had no systemic predisposing factors [20]. Furthermore, compared with controls, patients with xerostomia exhibited significantly increased presence of atrophy of tongue papillae and higher numbers of Candida [21]. The hematological examinations were not performed in the above-mentioned previous studies. Based on our findings and previous studies, we suggested that routine hematological screening for hematinic deficiencies should be assessed in all patients with AG. Further studies should investigate the association between oral candidiasis, xerostomia and vitamin B12 deficiency in the development of AG.

## Conclusion

AG may be an important clinical indicator of vitamin B12 deficiency, especially when the degree of tongue atrophy is more than 50% or complete atrophy is present. Moreover, we also suggest that vitamin B12 deficiency might be an etiological factor in AG and its grading.

## Data Availability

The datasets used and/or analysed during the current study available from the corresponding author on reasonable request.

## Declarations

### Author details

^1^ Department of Oral Medicine, Affiliated Stomatology Hospital of Guangzhou Medical University, Guangdong Engineering Research Center of Oral Restoration and Reconstruction, Guangzhou Key Laboratory of Basic and Applied Research of Oral Regenerative Medicine, Guangzhou, China.

## Abbreviations

AG: atrophic glossitis.
CI: Confidence Interval.
DNA: Deoxyribonucleic acid.
Hb: hemoglobin.
QR: Quartile range.
LCI: linked color imaging.
OLP: Oral lichen planus.
OR: Odds ratio.
OSF: Oral submucous fibrosis.
PV: pemphigus vulgaris.
RAS: Recurrent aphthous stomatitis.
